# Biocompatibility of Liposome Nanocarriers in the Rat Inner Ear After Intratympanic Administration

**DOI:** 10.1186/s11671-017-2142-5

**Published:** 2017-05-25

**Authors:** Jing Zou, Hao Feng, Rohit Sood, Paavo K. J. Kinnunen, Ilmari Pyykko

**Affiliations:** 1 Department of Otolaryngology Head and Neck Surgery, Center for Otolaryngology-Head and Neck Surgery of Chinese PLA, Changhai Hospital, Second Military Medical University, Changhai Road #168, 200433 Shanghai, China; 2 Hearing and Balance Research Unit, Field of Oto-laryngology, School of Medicine, University of Tampere, Tampere, Finland; 3 Helsinki Biophysics and Biomembrane Group, Department of Biomedical Engineering and Computational Sciences, Aalto University, Espoo, Finland; 4 Present Address: Department of Otorhinolaryngology/Head and Neck Surgery, University Medical Center Groningen, Groningen, The Netherlands

**Keywords:** Nanomaterial, Liposome, Drug Delivery, Inner Ear, Animal, Biological Response

## Abstract

Liposome nanocarriers (LPNs) are potentially the future of inner ear therapy due to their high drug loading capacity and efficient uptake in the inner ear after a minimally invasive intratympanic administration. However, information on the biocompatibility of LPNs in the inner ear is lacking. The aim of the present study is to document the biocompatibility of LPNs in the inner ear after intratympanic delivery. LPNs with or without gadolinium-tetra-azacyclo-dodecane-tetra-acetic acid (Gd-DOTA) were delivered to the rats through transtympanic injection. The distribution of the Gd-DOTA-containing LPNs in the middle and inner ear was tracked in vivo using MRI. The function of the middle and inner ear barriers was evaluated using gadolinium-enhanced MRI. The auditory function was measured using auditory brainstem response (ABR). The potential inflammatory response was investigated by analyzing glycosaminoglycan and hyaluronic acid secretion and CD44 and TLR2 expression in the inner ear. The potential apoptosis was analyzed using terminal transferase (TdT) to label the free 3′OH breaks in the DNA strands of apoptotic cells with TMR-dUTP (TUNEL staining). As a result, LPNs entered the inner ear efficiently after transtympanic injection. The transtympanic injection of LPNs with or without Gd-DOTA neither disrupted the function of the middle and inner ear barriers nor caused hearing impairment in rats. The critical inflammatory biological markers in the inner ear, including glycosaminoglycan and hyaluronic acid secretion and CD44 and TLR2 expression, were not influenced by the administration of LPNs. There was no significant cell death associated with the administration of LPNs. The transtympanic injection of LPNs is safe for the inner ear, and LPNs may be applied as a drug delivery matrix in the clinical therapy of sensorineural hearing loss.

## Background

Liposome nanocarriers (LPNs) are potentially the future of inner ear therapy due to their high drug loading capacity and efficient uptake in the inner ear after a minimally invasive intratympanic administration [1–4]. The intratympanic approach is well accepted by otologists as a rational targeted drug delivery approach because it avoids the unnecessary accumulation of therapeutic agents in non-targeted regions, which has been a prior strategy in the clinic for the treatments of Meniere’s disease and sudden sensorineural hearing loss using gentamicin and corticosteroids. The molecular targeting of model therapeutics in the cochlea was indicated by the intratympanic administration of specific peptide-functionalized LPNs [5]. Furthermore, the automatic sustained delivery of LPNs to the inner ear through the middle ear was achieved using a novel device composed of an osmotic pump and high-performance polyimide tubing [6]. As the oldest nanotherapeutic platform in the clinic, LPNs were safe in treating cancer, infectious disease, inflammation, pain, etc. [7–9]. However, the biocompatibility of LPNs in the middle and inner ears remains unknown and needs to be clarified before they can be applied clinically in otology.

**Fig. 1:**
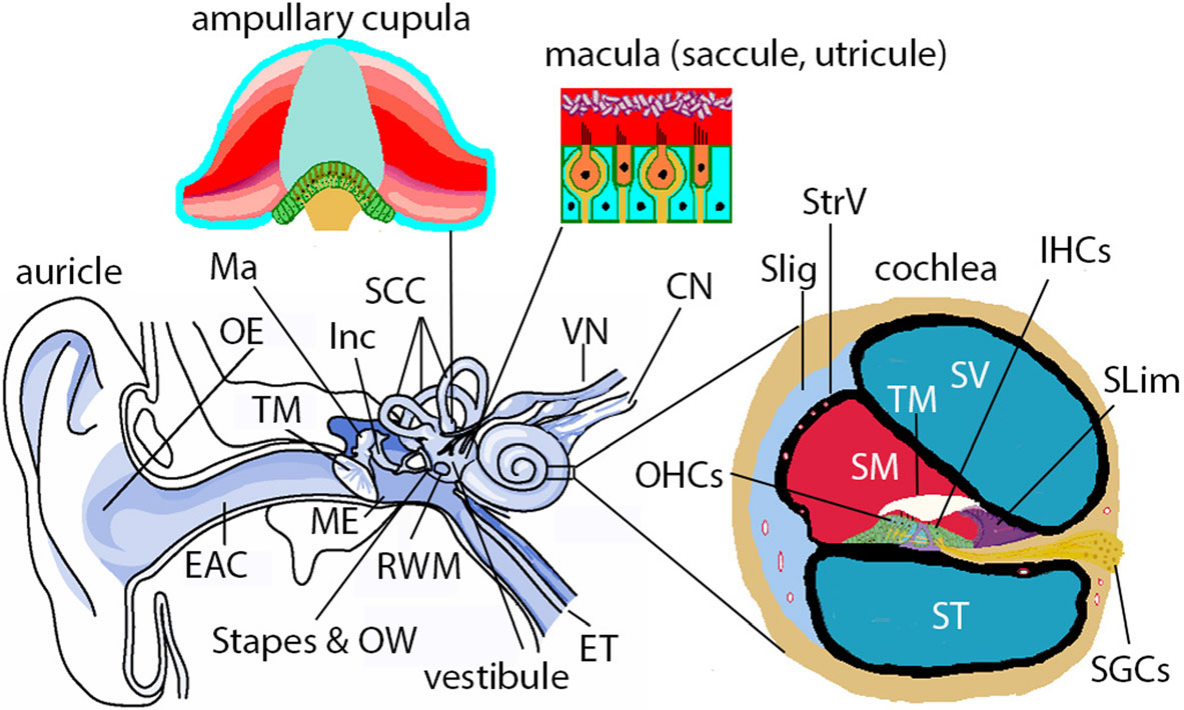
Illustration of the mammalian ear. The mammalian ear (including humans and rats) is composed of outer, middle, and inner ears. The outer ear (*OE*) is composed of auricle and external auditory canal (*EAC*). The middle ear (*ME*) is composed of the tympanic membrane (*TM*) and the cavity that houses the ossicular chain, including the malleus (*Ma*), incus (*Inc*), and stapes. The middle ear cavity is an extension of the nasopharynx via the Eustachian tube (*ET*) and communicates with the inner ear through the oval window (*OW*) and round window membrane (*RWM*). The inner ear is composed of cochlea and vestibular system. The cochlea is the sensory organ for hearing and has three chambers, i.e., the perilymphatic compartments of scala tympani (*ST*) and scala vestibuli (*SV*), and the endolymphatic compartment of scala media (*SM*). On the lateral wall of SM, there are the stria vascularis (*StrV*) and spiral ligament (*SLig*). On the bottom of SM, there are organ of Cortis that contains inner hair cells (*IHCs*) and outer hair cells (*OHCs*), tectorial membrane (*TM*), and spiral limbus (*Slim*). The spiral ganglion cells (*SGCs*) fire an action potential corresponding to the mechano-electrical transduction of the hair cells and supply all of the brain’s auditory input. The vestibular system is responsible for balance and is composed of three semicircular canals (*SCC*) and vestibule. The ampullary cupula within the SCC detect rotational accelerations and the macula within the saccule and utricule of the vestibule detect linear accelerations. *CN* cochlear nerve, *SP* spiral prominence, *VN* vestibular nerve, *VS* vas spiralis. (adapted from Zou J. Focal Drug Delivery in Inner Ear Therapy: in Focal Controlled Drug Delivery. Editors: Domb AJ and Khan W. Springer, London, UK. ISBN: 978-1-4614-9433-1, 2014; p215-224)

The ear is composed of external, middle, and inner ears (Fig. 1) that may be exposed to the LPNs after intratympanic delivery. The middle ear is the primary site that is exposed to the LPNs at their highest concentration, the inner ear is the therapeutic site and the most sensitive organ to hazardous agents, and the external ear canal has the potential to be irritated by outflowing agents from the middle ear cavity. Biological barriers are the first defense system, limiting the bioavailability of the agents, and exist in the skin, mucosa, and the perineural structures. The barrier system in the inner ear plays a critical role in maintaining the ionic homeostasis that is essential for the physiological activity of the inner ear. The functional alteration of these barriers can be accurately evaluated using gadolinium-enhanced magnetic resonance imaging (Gd-MRI). Impairment in the auditory function can be precisely measured through the auditory brainstem response (ABR). Therefore, the ear (including the external, middle, and inner ears) itself serves as an excellent model for nanotoxicology [10, 11].

Hyaluronic acid (hyaluronan) is a naturally occurring polyanionic biopolymer and is a primary component of the extracellular matrix in the basement membrane. Hyaluronic acid is composed of D-glucuronic acid and N-acetyl-D-glucosamine, which are linked via alternating β-1, 4 and β-1, 3 glycosidic bonds. The accumulation of hyaluronic acid might contribute to increased permeability and microcirculation inflammation in renal ischemic reperfusion injury [12]. The ototoxic effect of silver nanoparticles was shown to be correlated to the accumulation of hyaluronic acid in the rat cochlea in our previous report [11]. Hyaluronic acid binds to CD44 and toll-like receptor 2/4 (TLR2/4) in the tissue and triggers biological reactions [13, 14]. The biological activities mediated by CD44 upon binding to hyaluronic acid are mainly through interacting with regulatory and adaptor molecules, such as SRC kinases, Rho GTPases, VAV2, growth factor receptor-bound protein 2-associated-binding protein 1 (GAB1), ankyrin, and ezrin [15–17]. CD44 also mediates the metabolism of hyaluronic acid through the approaches of cellular uptake and degradation in addition to recruiting T cells to inflammatory sites and regulating T cell-mediated endothelial injury [18]. It was reported that the cytotoxicity to endothelial cells of the inner ear by anti-endothelial cell antibodies might play a role in causing the stria vascularis damage in immune-mediated sudden sensorineural deafness [19]. TLR2-dependent nuclear factor-κB activation was reportedly involved in non-typeable Haemophilus influenzae-induced monocyte chemotactic protein 1 upregulation in the spiral ligament fibrocytes of the inner ear, which might be the key step in inner ear dysfunction secondary to chronic otitis media [20]. If LPNs induce inner ear impairment after middle ear administration, the TLR2-mediated signaling pathway should be the important mechanism.

 We aimed to evaluate the biocompatibility of LPNs in the inner ear after transtympanic injection. The functions of the biological barriers in the skin (external ear canal), mucosa (middle ear cavity), and inner ear compartments were measured using Gd-MRI at various time points. The auditory function was evaluated using ABR measurement. Finally, the potential histopathological changes were analyzed by measuring the accumulations of glycosaminoglycans and hyaluronic acid, the expressions of CD44 and TLR2, and DNA fragmentation in the cochlea.

## Results

### LPNs did not Cause Functional Changes in Rat Cochlea

**Fig. 2:**
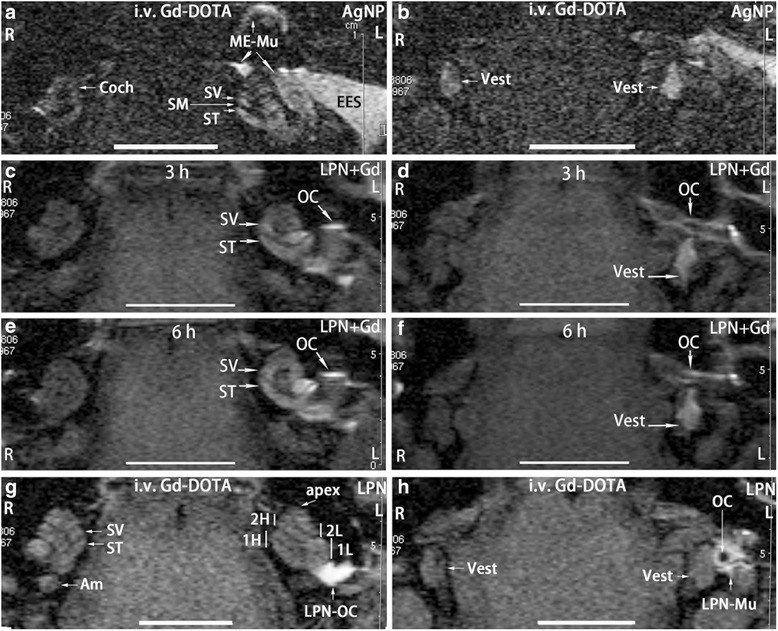
Gadolinium-enhanced MRI of rat inner ear after liposome nanocarrier (LPN) administration. In all animals, nanomaterials were injected onto the medial wall of left middle ear cavity. The positive control was imaged in rats at 2 h post-intravenous injection of Gd-DOTA secondary to transtympanic injection of silver nanoparticles (AgNPs) 5 h in advance (**a**, **b**). Dynamic distribution of LPNs in the middle and inner ears was shown in **c**, **d**, **e**, **f** by transtympanic injection of Gd-DOTA-containing LPN without intravenous administration of Gd-DOTA. The impact of empty LPNs on the biological barrier was shown by MRI at 2 h post-intravenous injection of Gd-DOTA (i.v. Gd-DOTA) in rats receiving transtympanic injection of LPN 5 h in advance (**g**, **h**). *Am* ampullar of posterior semicircular canal, *Coch* cochlea, *EES* external ear skin, *L* left ear, *LPN-Mu* LPN in the middle ear mucosa, *LPN-OC* LPN on the ossicular chain (*OC*), *ME-Mu*, middle ear mucosa, *R* right ear, *SM scala media*, *ST* scala tympani, *SV* scala vestibuli, *Vest* vestibulum, *1H* basal higher turn of cochlea, *1L* basal lower turn of cochlea, *2H* second higher turn of cochlea, *2L* second lower turn of cochlea. *Scale bar* = 5 mm

In the positive control group, bright signal in the perilymph of cochlea (Coch) and the vestibular (Vest) on both sides (L, R) (Fig. 2a, b) indicating uptake of Gd-DOTA. After transtympanic injections of silver nanoparticles (AgNPs), the signal intensities in the perilymphatic compartments significantly increased while extremely intense signal was also detected in the external ear canal skin, middle ear mucosa, indicating the enhanced uptake of Gd-DOTA associated with AgNP administration (L in Fig. 2a, b) (Table 1). The evaluation system was therefore validated. In the animal receiving transtympanic injection of LPN + Gd-DOTA, bright signal was detected on the surface of ossicular chain, scala vestibuli, scala tympani, and vestibule at 3 h post-injection indicating obvious distribution of LPN in these regions (Fig. 2c, d). The signal intensity in the scala vestibuli in the basal turn was visibly stronger than that in the scala tympani suggesting an efficient entry of LPN through the oval window in the current animal [21]. At 6 h post-injection, the signal intensities between the scala vestibuli and scala tympani in the basal turn became similar and the whole cochlea showed almost homogenous signal, but there was insignificant changes in the vestibule (Fig. 2e, f). In animals receiving intravenous injections of Gd-DOTA following transtympanic injection of blank LPNs, both sides displayed similar signal intensities except that there were strong signals in the middle ear receiving transtympanic injection of blank LPNs suspecting accumulation of LPNs on the surface of ossicular chain (Fig. 2g, h). The black hole in the ossicular chain indicating the hollow area of the stapes (Fig. 2h). Equal signal intensities on both sides suggested that the transport property for Gd-DOTA of the blood-perilymph barriers on both ears did not change after transtympanic injection of LPNs (Fig. 2g, h) (Table 1).

**Table 1:** Signal ratio of the inner ear region of interest in gadolinium-enhanced MRI

ID of rats	treatment	Signal ratio of treatment over untreated control in the region of interest		
		ST	SV	Vest
281	LPNs	1.04	0.93	1.01
282	LPNs	0.99	0.95	1.01
269	AgNPs	1.30	1.37	1.29
271	AgNPs	1.36	1.03	1.05

Gd-DOTA (0.725 mM/kg) was injected into the tail vein 2 h before the MRI measurements. *AgNPs* silver nanoparticles, *ID* identification number, *LPNs* liposome nanocarriers, *ST* scala tympani, *SV* scala vestibuli, *Vest* vestibulum

**Fig. 3:**
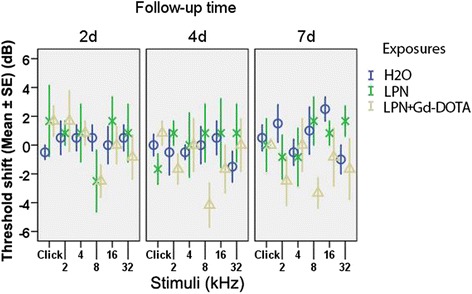
Impact of transtympanic injection of liposome nanocarriers on hearing function in rats measured by the auditory brainstem response. Hearing loss was expressed as threshold shifts. There was insignificant difference among groups (*p* > 0.05, one-way ANOVA). *n* = 6 in each group. *H2O* transtympanic injection of deionized water in negative control group, *LPN* empty liposome nanocarrier, *LPN + Gd-DOTA* Gd-DOTA-containing LPN, *2d*, *4d*, *and 7d* 2, 4, and 7 days after injection

Neither LPN + Gd-DOTA nor LPNs caused significant hearing loss, presented as an ABR threshold shift that was measured using stimuli of click and tone bursts at the frequencies of 2, 4, 8, 16, and 32 kHz at 2, 4, and 7 days post-administration, compared to the ears receiving transtympanic injections of deionized water (dH_2_O) (Fig. 3).

### LPNs did not Induce Glycosaminoglycan Accumulation in Rat Cochlea

**Fig. 4:**
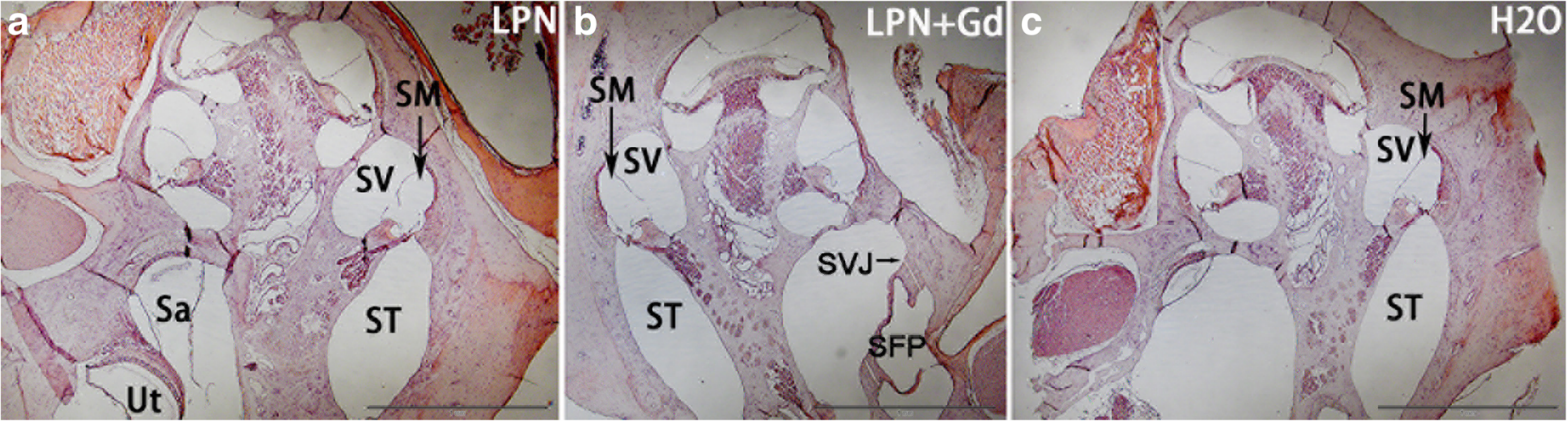
Hematoxylin-eosin staining of rat cochleae exposed to liposome nanocarriers. There was no inflammatory infiltration in the cochlea received administrations of LPN (**a**), LPN+Gd (**b**), and H2O (**c**). *Circled area* indicated selection of region of interests for intensity measurements (**a**). *LPN* empty liposome nanocarrier, *LPN + Gd* Gd-DOTA-containing LPN. *Sa* saccule, *SFP* stapes footplate, *SVJ* stapediovestibular joint, *SM* scala media, *ST* scala tympani, *SV* scala vestibuli, *Ut* utricule. *Scale bar* = 1 mm

**Fig. 5:**
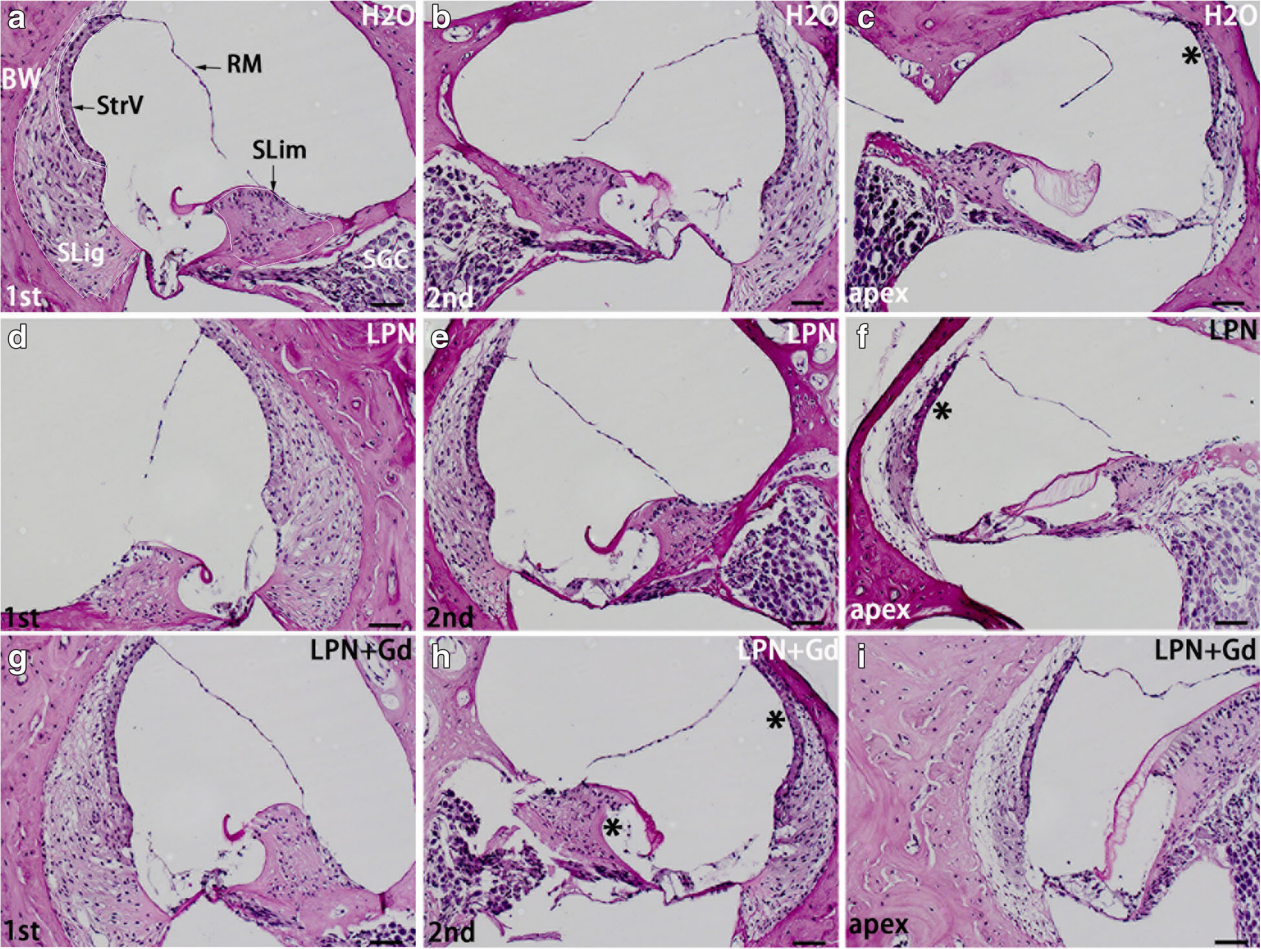
Glycosaminoglycan secretion in rat cochlea exposed to liposome nanocarriers was detected using periodic acid Schiff’s staining light microscopy. The spiral limbus (*SLim*) and bony wall (*BW*) of the cochlea showed the most intensive staining in groups of negative control (*H2O*) (**a**–**c**), empty liposome nanocarrier (LPN) (**d**–**f**), and Gd-DOTA-containing LPN (*LPN + Gd*) (**g**–**i**). The staining area with visibly higher intensities were indicated by * in **c**, **f**, and **h** in comparison to the left column. *RM* Reissner’s membrane, *SGC* spiral ganglion cell, *SLig* spiral ligament, *StrV* stria vascularis, *1st* basal turn, *2nd* second turn. *Scale bar* = 50 μm

**Fig. 6:**
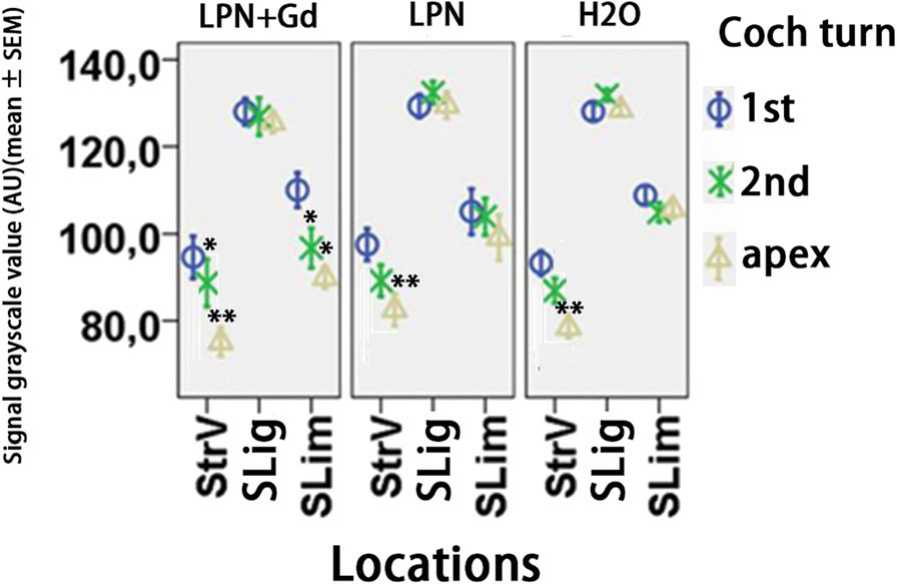
Quantification of glycosaminoglycan secretion in rat cochlea exposed to liposome nanocarriers detected using periodic acid Schiff’s staining. *n* = 3 in each group. *AU* arbitrary unit, *H2O* negative control, *LPN* empty liposome nanocarrier, *LPN + Gd* Gd-DOTA-containing LPN, *SLig* spiral ligament, *Slim* spiral limbus, *StrV* stria vascularis, *1st* basal turn, *2nd* second turn. *p < 0.05, **p < 0.01 (one-way ANOVA with LSD test used as post hoc analysis)

Hematoxylin and eosin staining did not demonstrate any inflammatory infiltration of leukocyte and fibrin in the cochlea of all analyzed animals including the stapes and oval window where the LPNs pass through (Fig. 4). Periodic acid Schiff’s staining demonstrated the existence of glycosaminoglycans in the bony wall, spiral limbus, spiral ligament, tectorial membrane, Reissner’s membrane, osseous spiral lamina, and stria vascularis in the cochlea of animals receiving transtympanic injections of dH_2_O. There was a gradient increase in the signal intensity from the basal turn to the apex, and the difference was significant in the stria vascularis (Figs. 5 and 6). The signal gradient in the cochlea was not changed in the animals receiving transtympanic injection of LPNs and LPN + Gd-DOTA (Figs. 5 and 6).

### There was Minor Impact on the Hyaluronic Acid Secretion in Rat Cochlea by LPNs

**Fig. 7:**
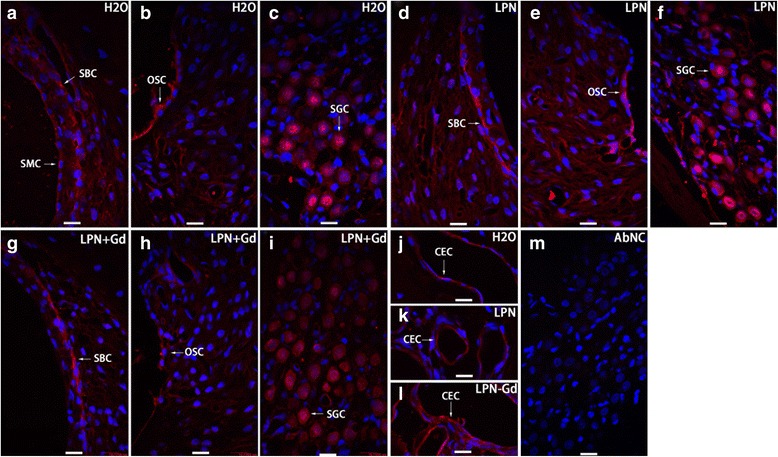
Hyaluronic acid secretion in rat cochlea exposed to liposome nanocarriers was detected with immunofluorescent confocal microscopy. Positive staining was found in the stria basal cell (*SBC*), outer sulcus cell (*OSC*), spiral ganglion cell (*SGC*), and capillary endothelial cell (*CEC*) of modiolus of groups of negative control (*H2O*) (**a**–**c**, **j**), empty liposome nanocarriers (*LPN*) (**d**–**f**, **k**), and Gd-DOTA-containing LPN (*LPN + Gd*) (**g**–**i**, **l**). There was no staining in the antibody omitted negative control (*AbNC*) (**m**). *CEC* capillary endothelial cell, *ISC* inner sulcus cell, *SBC* stria basal cell, *SL-I* spiral ligament fibrocyte type I, *SLSF* spiral limbus satellite fibrocyte, *SMC* stria vascularis marginal cell. *Scale bar* = 16 μm

**Fig. 8:**
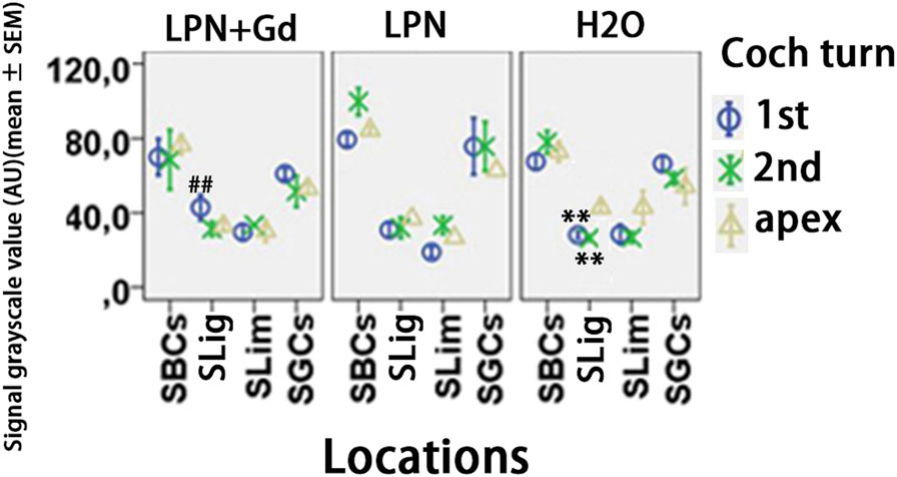
Quantification of hyaluronic acid secretion in rat cochlea exposed to liposome nanocarriers detected using immunofluorescent confocal microscopy. *n* = 3 in each group. *AU* arbitrary unit, *H2O* negative control, *LPN* empty liposome nanocarrier, *LPN + Gd* Gd-DOTA-containing LPN, *SBCs* stria basal cells, *SGCs* spiral ganglion cells, *SLig*, spiral ligament, *Slim* spiral limbus, *1st* basal turn, *2nd* second turn. **p < 0.01 (comparing to the apex), ##*p* < 0.01 (comparing to LPN and H2O groups of the basal turn) (one-way ANOVA with LSD test used as post hoc analysis)

In the cochlea of rats receiving transtympanic injections of dH2O, positive staining for hyaluronic acid was detected predominantly in the spiral ganglion cells, strial basal cells, outer sulcus cells, and capillary endothelial cells, among other cells (Fig. 7). The signal intensities in the spiral ligament fibrocytes of the basal and second turns were significantly higher than that of the apex. These differences became insignificant in the cochlea of rats with the application of LPNs and LPN + Gd-DOTA, indicating that the secretion of hyaluronic acid by the spiral ligament fibrocytes was affected by the LPN administration (Fig. 8). LPN + Gd-DOTA also reduced staining in the spiral ligament fibrocytes of the basal turn. However, there was no impact on the secretion of hyaluronic acid in the majority of the cochlear cells by the transtympanic injection of LPNs and LPN + Gd-DOTA (Figs. 7 and 8).

### LPNs did not Alter the CD44 Cell Population in the Rat Cochlea

**Fig. 9:**
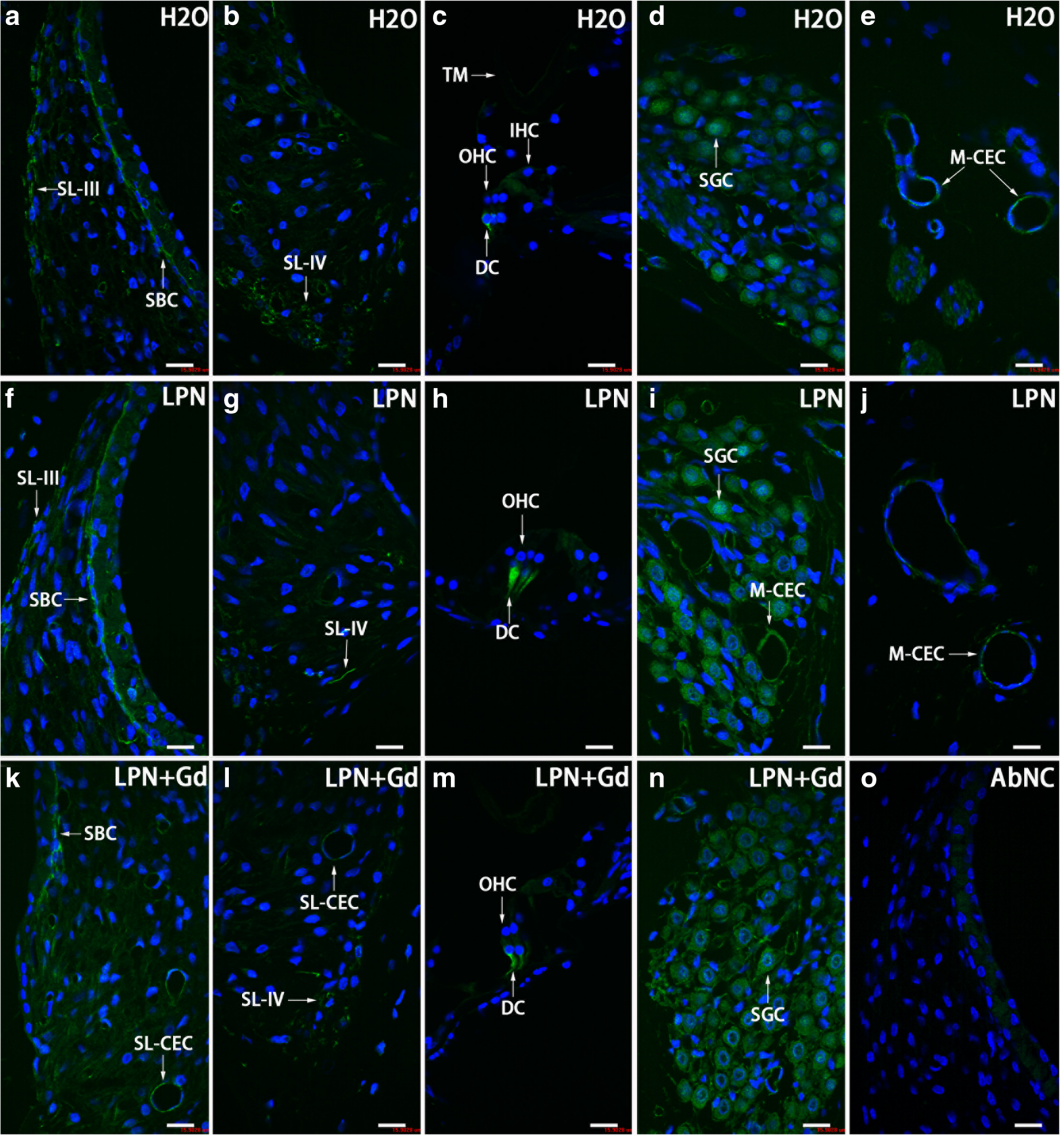
CD44-positive cell distribution in the rat cochlea exposed to liposome nanocarrier demonstrated using immunofluorescent confocal microscopy. CD44-positive cells were mainly detected in the stria basal cell (*SBC*), spiral ligament (*SL*), Dieter’s cells (*DC*), spiral ganglion cell (*SGC*), and capillary endothelial cell (*CEC*) in the groups of negative control (*H2O*) (**a**–**e**), empty liposome nanocarriers (*LPN*) (**f**–**j**), and Gd-DOTA-containing LPN (*LPN + Gd*) (**k**–**n**). There was no staining in the antibody omitted negative control (*AbNC*) (**o**). *IHC* inner hair cells, *M-CEC* capillary endothelial cell in modiolus, *SL-CEC* capillary endothelial cell in spiral ligament: spiral ganglion cells, *SL-III* spiral ligament fibrocyte type III, *SL-IV* spiral ligament fibrocyte type IV, *TM* tectorial membrane, *OHC* outer hair cells. *Scale bar* = 16 μm

**Fig. 10:**
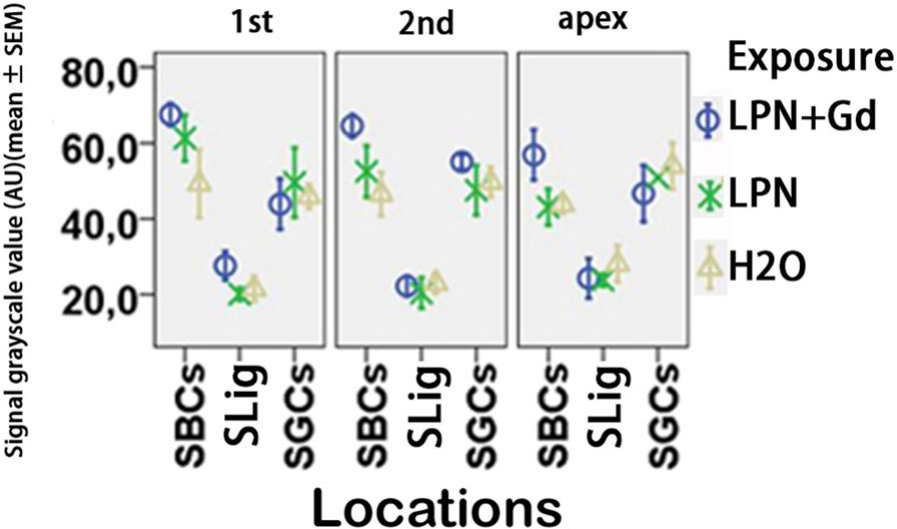
Quantification of CD44 protein level in rat cochlea exposed to liposome nanocarriers detected using immunofluorescent confocal microscopy. There was insignificant difference among groups (*p* > 0.05, one-way ANOVA). *n* = 3 in each group. *n* = 3 in each group. *AU* arbitrary unit, *H2O* negative control, *LPN* empty liposome nanocarrier, *LPN + Gd* Gd-DOTA-containing LPN, *SBCs* stria basal cells, *SGCs* spiral ganglion cells, *SLig* spiral ligament, *1st* basal turn, *2nd* second turn

In the cochleae exposed to dH_2_O, the strial intermediate cells, strial basal cells, spiral ligament fibrocytes, spiral ganglion cells, Deiters’ cells in the organ of Corti, and capillary endothelial cells in the modiolus and spiral ligament showed intensive staining for CD44. There was an insignificant difference in the signal intensities among the cochlear turns. The CD44-positive population and expression intensity were not affected by the transtympanic injection of either LPN + Gd-DOTA or LPNs (Figs. 9 and 10).

### LPNs did not Alter TLR2 Expression in the Rat Cochlea

**Fig. 11:**
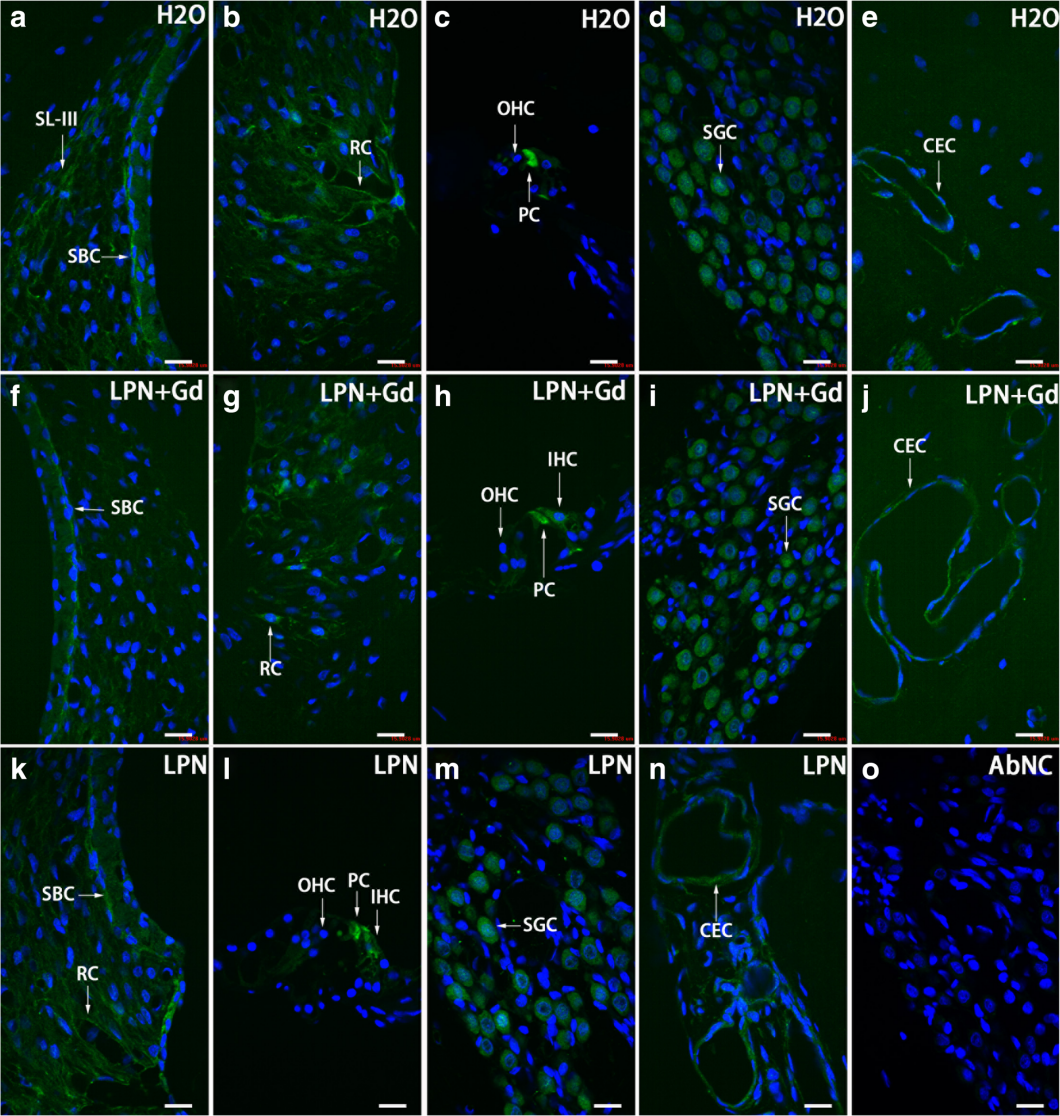
TLR2-positive cell distribution in the rat cochlea exposed to liposome nanocarrier demonstrated using immunofluorescent confocal microscopy. TLR2-positive cells were mainly detected in the stria basal cell (*SBC*), spiral ligament (*SL*), root cell (*RC*), pillar cell (*PC*), spiral ganglion cell (*SGC*), and capillary endothelial cell (*CEC*) in the groups of negative control (*H2O*) (**a**–**e**), empty liposome nanocarriers (*LPN*) (**k**–**n**), and Gd-DOTA-containing LPN (*LPN + Gd*) (**f**–**j**). There was no staining in the antibody-omitted negative control (*AbNC*) (**o**). *IHC* inner hair cells, *SL-III* spiral ligament fibrocyte type III, *OHC* outer hair cells. *Scale bar* = 16 μm

**Fig. 12:**
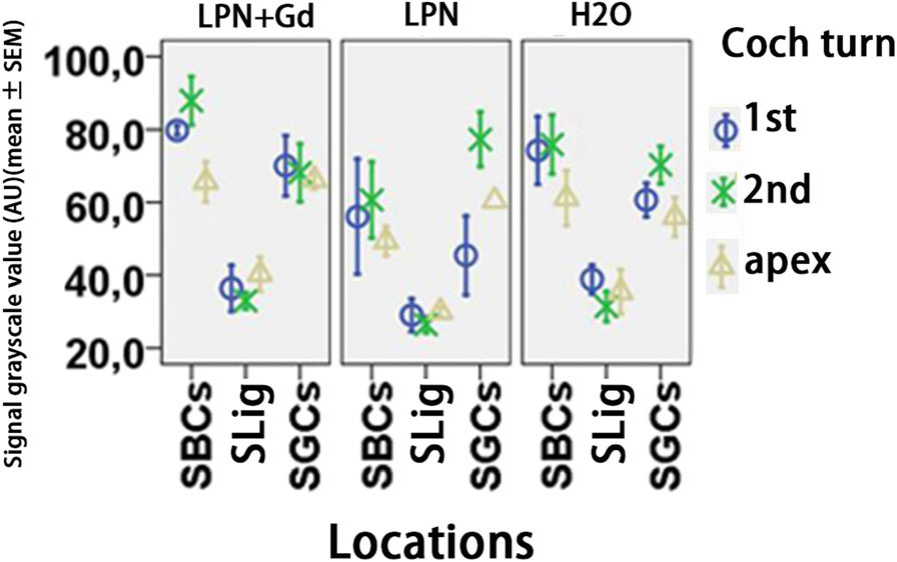
Quantification of TLR2 protein level in rat cochlea exposed to liposome nanocarriers detected using immunofluorescent confocal microscopy. There was insignificant difference among groups (*p* > 0.05, one-way ANOVA). *n* = 3 in each group. *AU* arbitrary unit, *H2O* negative control, *LPN* empty liposome nanocarrier, *LPN + Gd* Gd-DOTA-containing LPN, *SBCs* stria basal cells, *SGCs* spiral ganglion cells, *SLig* spiral ligament, *1st* basal turn, *2nd* second turn

In the cochleae exposed to dH2O, the strial basal cells, spiral ligament fibrocytes, root cells, spiral ganglion cells, pillar cells of the organ of Corti, and capillary endothelial cells in the modiolus showed intensive staining for TLR2. There was an insignificant difference in the signal intensities among the cochlear turns. The TLR2-positive population and expression intensity were not affected by the transtympanic injection of either LPN + Gd-DOTA or LPNs (Figs. 11 and 12).

### LPNs did not Cause Cell Death in Rat Cochlea

**Fig. 13:**
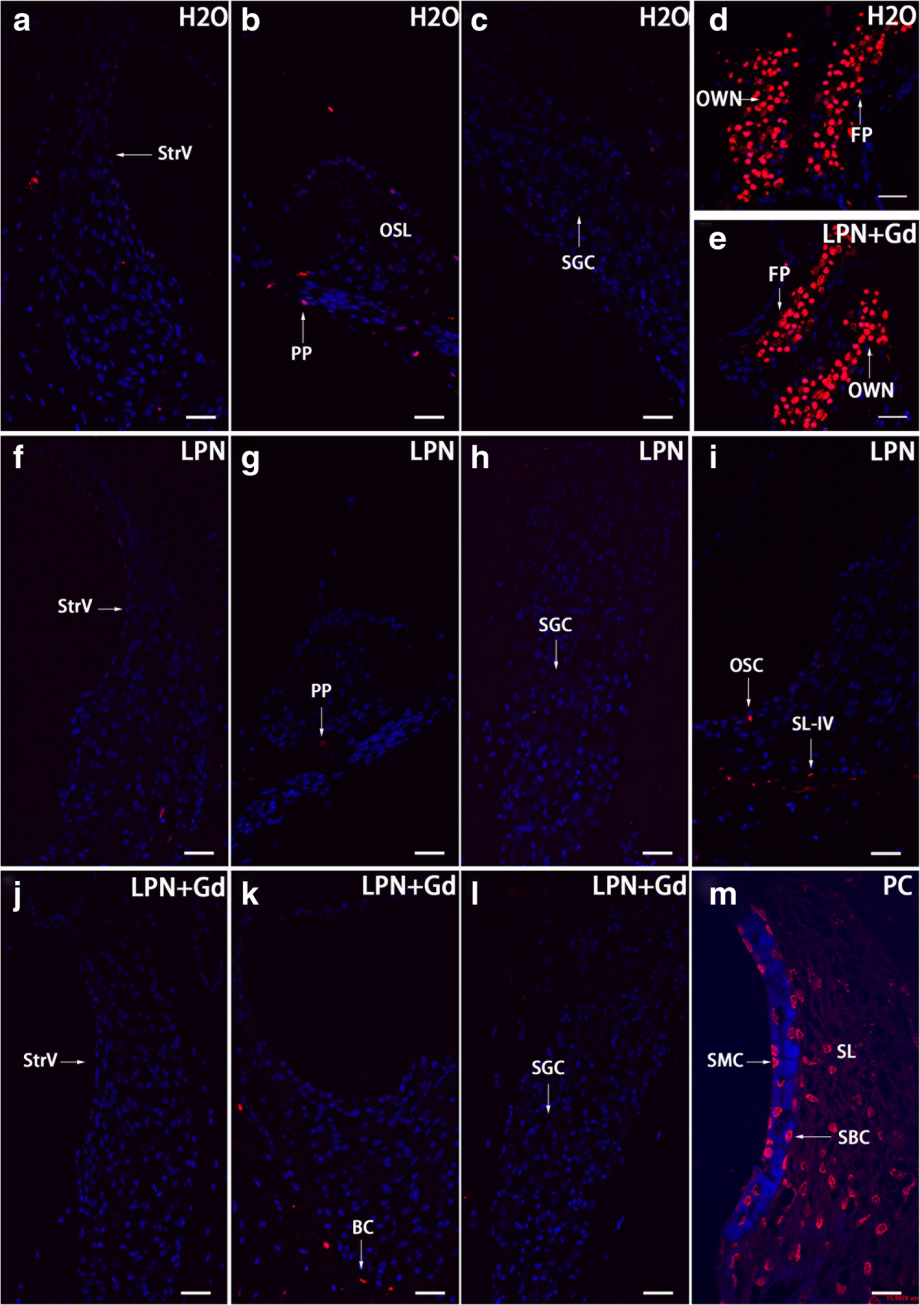
Apoptosis in the rat cochlea exposed to liposome nanocarriers demonstrated using TUNEL staining confocal microscopy. Apoptotic cells were sparsely detected in the cochlear cells of rats in groups of negative control (*H2O*) (**a**–**c**), empty liposome nanocarriers (*LPN*) (**f**–**i**), and Gd-DOTA-containing LPN (*LPN + Gd*) (**j**–**l**). There were abundant apoptotic cells in the footplate of the stapes (*FP*) and oval window niche (*OWN*) of both groups (**d**, **e**). In a positive control (PC), abundant TUNEL staining was detected in the spiral ligament fibrocytes (*SL*), stria basal cells (*SBC*), and stria margina cells (*SMC*). *PP* periphery process of the spiral ganglion cell (*SGC*), *OC* osteocyte, *OSC* outer sulcus cell, *SL-IV* type IV of spiral ligament fibrocytes, *SLim* spiral limbus, *StrV* stria vascularis. *Scale bar* **a**–**l** = 32 μm, **m** = 16 μm

There were sparse apoptotic cells that are randomly distributed in the cochlea of non-treated rats. Surprisingly, there were abundant apoptotic cells in the footplate of the stapes and oval window niche. There was no impact on the amount and distribution pattern of apoptotic cells by the administration of LPNs and LPN + Gd-DOTA (Fig. 13).

## Discussion

LPNs entered the inner ear efficiently after transtympanic injection demonstrated by MRI using Gd-DOTA as drug mimetics that were encapsulated inside the LPNs (Fig. 2c–f). Although a previous study showed that the round window was the major pathway of LPNs to enter the inner [6], the present observation displayed that the oval window pathway was more efficient than the round window to transport the LPNs from the middle ear into the inner ear. This result suggested that both pathways are important in the inner ear loading of LPNs after targeted middle ear medial wall administration. Using the most efficient in vivo method of gadolinium-enhanced inner ear MRI to evaluate the biological barrier and frequency-specific ABR to assess the hearing function, the present study demonstrated that transtympanic injection of LPNs and LPN + Gd-DOTA neither disrupted the function of the inner ear barriers nor caused hearing impairment in rats. By analyzing the previously demonstrated critical inflammatory biological markers [11, 22], LPNs and LPN + Gd-DOTA did not induce the inflammatory response in the cochlea. Although the round window membrane was not evaluated, absence of inflammation in the stapes and oval window ruled out an obvious inflammatory reaction in the round window membrane since the present study demonstrated that the oval window pathway was superior to the round window approach for LPNs.

Inner ear MRI after the intravenous injection of gadolinium chelate is capable of detecting the oxidative stress-mediated disruption in the blood-perilymph and blood-endolymph barriers induced by mitochondrial toxins [23]. AgNPs were reported to cause cellular impairment through the generation of reactive oxygen species (ROS) and the activation of Jun amino-terminal kinases (JNK), leading to the release of cytochrome C into the cytosol and the translocation of Bax to the mitochondria [24]. In transtympanic injection, AgNPs entered the inner ear and induced permeability changes in the biological barriers of the rat inner ear [11, 25]. LPNs also entered the rat inner ear after the transtympanic injection in a size-dependent pattern, and the 95 nm-diameter LPNs showed the highest efficacy in passing through the middle-inner ear barriers [3]. In the present study, the mean size of LPNs was 100 to 115 nm, which was slightly bigger than the most efficient size. LPN + Gd-DOTA showed that this size of LPNs entered the inner ear, which is in accordance with the previous report [3]. However, the entry of LPNs into the inner ear did not cause permeability changes in the blood-perilymph and blood-endolymph barriers. This result suggested that LPNs are safe for the inner ear. ABR results indicating a normal hearing function supported the MRI result.

There was an association between hyaluronic acid secretion and permeability change and microcirculation inflammation in renal ischemic reperfusion injury [12]. The previous study also showed that AgNPs caused the accumulation of hyaluronic acid in the rat cochlea [11]. CD44 and toll-like receptor 2/4 (TLR2/4) work as receptors of hyaluronic acid and trigger biological reactions [13, 14]. CD44 also mediates the metabolism of hyaluronic acid through cellular uptake and degradation in addition to recruiting T cells to inflammatory sites and regulating T cell-mediated endothelial injury [18]. In the present study, hyaluronic acid, CD44, and TLR2 were detected in the rat cochlea. LPN + Gd-DOTA reduced the secretion of hyaluronic acid in the spiral ligament fibrocytes. The expressions of CD44 and TLR2 were not changed after the transtympanic injection of either LPNs or LPN + Gd-DOTA. The total glycosaminoglycan, which contains hyaluronic acid in the cochlea was not affected by the administrations of LPNs and LPN + Gd-DOTA. The impact of LPNs on the hyaluronic acid distribution in rat cochlea did not cause either permeability change or hearing loss, indicating that the modification is unharmful. It was reported that macrophages undergo phenotypic changes dependent on molecular weight of hyaluronan that correspond to either pro-inflammatory response for low molecular weight hyaluronic acid or anti-inflammatory response for high molecular weight hyaluronic acid [26]. The observed minor changes of hyaluronic acid distribution in the cochlea might have high molecular weight and anti-inflammatory function. Therefore, there was no hint of an inflammatory reaction in the rat cochlea.

The observed apoptosis in the stapes footplate cells might be normal biological activity. A balance between survival and apoptosis in the stapes footplate cells was reportedly as necessary to inactivate the otosclerosis [27]. Administration of LPN + Gd-DOTA did not affect apoptosis in the rat stapes.

## Conclusions

The present study demonstrated that the transtympanic injection of liposome nanocarriers neither impaired the biological barriers of the inner ear nor caused hearing loss in the rats. The critical inflammatory mechanism was not activated by the administration of liposome nanocarriers, either. The results suggested that transtympanic injection of liposome nanocarrier is safe for the cochlea of rat.

## Methods

### Materials

Sphingosine (Sph), 1-stearoyl-2-oleoyl-sn-glycero-3-phosphocholine (SOPC), and 1, 2-distearoyl-sn-glycero-3-phosphoethanolamine-N-[methoxy(polyethyleneglycol)-2000] (ammonium salt) [DSPE-PEG-2000] were purchased from Avanti polar lipids (Alabaster, USA). DiI (Vybrant DiI cell-labeling solution, 1 mM in solvent) and N-(6-tetramethylrhodaminethiocarbamoyl)-1,2-dihexadecanoyl-sn-glycero-3-hosphoethanolamine, triethylammonium salt (TRITC-DHPE) were purchased from Thermo Fisher Scientific (Waltham, USA). Gd-DOTA (DOTAREM) was from Guerbet, Cedex, France. Hepes and EDTA were from Sigma. The purity of the lipids was evaluated using thin-layer chromatography on silicic acid-coated plates (Merck, Darmstadt, Germany) developed with a chloroform/methanol/water mixture (65:25:4, v/v/v). An examination of the plates after iodine staining and, when appropriate, upon UV illumination revealed no impurities. The lipid concentrations were determined gravimetrically with SuperG (Kibron, Espoo, Finland); a high-precision microbalance. The polyvinylpyrrolidone-stabilized AgNPs were supplied by Colorobbia (Firenze, Italy). The AgNPs were dispersed in deionized water (370.7 mM), and scanning electron microscopy showed that the AgNPs are spheroids with a particle size of around 100 nm. Dynamic light scattering (DLS) showed a mean hydrodynamic size of 117 ± 24 nm and a mean zeta potential of −20 ± 9 mV.

In the visualization of nanocarrier uptake in the inner ear and the evaluation of biological barrier function, 5 male Sprague Dawley rats, weighing between 334 and 348 g, were provided by the Biomedicum Helsinki, Laboratory Animal Centre, University of Helsinki, Finland (this is the defined animal center that provides animals for MRI experiments in Biomedicum); in the ABR and histological studies, 18 Sprague Dawley rats, weighing between 300 and 400 g, were provided by the Experimental Animal Unit of the University of Tampere School of Medicine in Finland. Animal assignments in each study were shown in Table 2. All animal experiments were approved by the Ethical Committee of University of Tampere (permission number: ESAVI/3033/04.10.03/2011). Animal care and experimental procedures were conducted in accordance with European legislation. Animals in the Gd-MRI study were anesthetized with isoflurane with 5% isoflurane–oxygen mixture (flow-rate 1.0 L/min) for induction and 3% for maintenance via a facemask. Animals for the ABR and histological studies were anesthetized with a mixture of 0.5 mg/kg medetomidine hydrochloride (Domitor^®^, Orion, Espoo, Finland) and 75 mg/kg ketamine hydrochloride (Ketalar^®^, Pfizer, Helsinki, Finland) via intraperitoneal injection followed by an intramuscular injection of enrofloxacin (Baytril^®^vet, Orion, Turku, Finland) at a dose of 10 mg/kg to prevent potential infection. The animal’s eyes were protected by Viscotears® (Novartis Healthcare A/S, Copenhagen, Denmark).

**Table 2:** Assignments of rats in MRI and ABR measurements post-intratympanic administration of liposome nanocarriers

Measurements	Number of ears			
	LPNs	LPN + Gd-DOTA	AgNPs	NC
MRI	2	1	2	5^a^
ABR	6	6		6^a^
HE staining	6	6		6^a^
Schiff’s staining	6	6		6^a^
Hyaluronic acid	3	3		6^a^
TLR2	3	3		6^a^
CD44	3	3		6^a^
TUNEL staining	6	6		6^a^

*ABR* auditory brainstem response, *HE* hematoxylin-eosin, *LPNs* liposome nanocarriers, *LPN + Gd-DOTA* Gd-DOTA-containing LPN, *NC* negative control, *TLR* toll-like receptor, *TUNEL* terminal deoxynucleotidyl transferase dUTP nick end labeling. ^a^The contralateral ears were used as negative controls in all studies

### Preparation and Characterization of LPNs with and without Gd

#### Preparation of Gd-containing LPNs

LPNs of unilamellar vesicles with an apparent hydrodynamic particle diameter (*Z*_av_) of 110 ± 15 nm that contain Gd-DOTA were prepared according to the previously published method [6]. A concentration of 1 mM Gd-DOTA-containing LPN (LPN + Gd-DOTA) refers to 1 mM liposomes encapsulating 500 mmol/L of Gd-DOTA.

#### Preparation of Blank LPNs

Blank LPNs of unilamellar vesicles with *Z*_av_ of 115 ± 10 nm were prepared according to a previous publication [6].

### Administration of LPNs

Under general anesthesia with isoflurane with 5% isoflurane–oxygen mixture (flow-rate 1.0 L/min), 50 μl of either LPNs or LPN + Gd-DOTA were injected into the left middle ear cavity through the tympanic membrane penetration under an operating microscope (OPMI1-F, Carl Zeiss, Jena, Germany). The same amount of deionized water (H_2_O) was injected transtympanically in rats that were assigned to the negative group. After the injection, the animals were kept in the lateral position with the injected ear oriented upward for 15 min before further measurements.

### Evaluation on Biological Barrier Function Using Gd-MRI

One animal receiving transtympanic injection of LPN + Gd-DOTA was selected to demonstrate distributions of LPN in the inner ear using MRI without contrast agent. Two animals receiving transtympanic injection of blank LPNs were engaged in MRI study for evaluation of the biological barrier function. Two animals receiving transtympanic injection of AgNPs (370.7 mM, 40 μl) were used as positive control. The contralateral ear without any injection was used as negative control in all studies. A 4.7T MR scanner with a bore diameter of 155 mm (PharmaScan, Bruker BioSpin, Ettlingen, Germany) was utilized. The maximum gradient strength was 300 mT/m with an 80-μs rise time. A gadolinium-tetraazacyclododecane-tetraacetic acid (Gd-DOTA, 500 mM, DOTAREM, Guerbet, Cedex, France) solution was injected into the tail vein (0.725 mM/kg) 2 h before the MRI measurements. The imaging protocol and rapid acquisition with relaxation enhancement (RARE) sequences were applied according to a previous publication [10]. MRI scanning commenced at several time points after the transtympanic injection. The first MRI time of around 5 h was determined by taking the penetration time of liposome nanoparticles from the middle ear to the inner ear as a reference [1, 3, 6]. The final imaging time of 8 d was selected according to the course of potential acute inflammation and the availability of the scanner. ParaVision PV 4.0 (Bruker, MA, USA) software was used for the post-processing and quantification of MR images.

### ABR Measurement

The auditory function of animals receiving injections of both blank and Gd-containing LPNs were evaluated using ABR measurements using BioSig32 (Tucker Davis Technologies, FL, USA) in a custom made, soundproof chamber. The ABR thresholds upon click and tone burst stimuli were recorded before and at a certain time point post-administration of LPNs. The first ABR measurement was followed on 2 days post-administration of AgNPs, allowing the animals to recover from the general anesthesia during the injection and to ensure the injected solution to be entirely cleared from the middle ear cavity. The second follow-up time of 4 days post-injection was chosen because it is close to the peak time of potential mitochondrial impairment-induced cell death in the cochlea [22]. The third follow-up time of 7 days is the time point when temporary threshold shifts remained significantly approved in an animal model of mitochondrial toxin-induced hearing loss [28]. The ABR recording procedure followed the previous report [11].

### Glycosaminoglycan Staining in Rat Cochlea After Administration of LPNs

Hematoxylin-eosin staining to assess potential inflammatory infiltration and periodic acid Schiff’s staining to evaluate potential glycosaminoglycan accumulation in the cochlea after administration of LPNs were performed according to a previous publication after ABR measurements over 7 days [11] The slices were observed and digital images were acquired under a light microscope (Leica DM2000 microscope equipped with an Olympus DP25 camera) for further analysis.

### Immunofluorescence Staining for Hyaluronic Acid and Receptors

Immunofluorescence staining for hyaluronic acid, CD44, and TLR2 were performed according to a previous publication after ABR measurements over 7 days [11, 21].

### Cell Death Detection

Potential nuclear DNA fragmentation in the cochlea was investigated using terminal transferase (TdT) to label the free 3′OH breaks in the DNA strands of apoptotic cells with TMR-dUTP (TUNEL staining) following the reported procedure [11]. Slices exposed to recombinant DNase I (Fermentas, Vantaa, Finland, 100 U/ml in 50 mM Tris/HCl, pH 7.5, 1 mg/ml bovine serum albumin) at 37 °C for 10 min, which induced DNA strand breaks prior to the labeling procedures, were utilized as positive controls. The samples were observed under a confocal microscope.

### Confocal Microscopy

The samples were observed under a Nikon inverted microscope (ECLIPSE Ti) combined with an Andor confocal system installed with Andor iQ 2.8 software (Andor Technology, Belfast, UK). The excitation lasers were 488 nm (green excitation) and 568 nm (red excitation) from an Andor laser combiner system, and the corresponding emission filters were 525/50 (Alexa Fluor-488) and 607/45 nm (Cy^TM^3 and TMR Red). DAPI was excited with light at 405 nm generated from a light-emitting diode and was detected using a 450–465 nm filter.

### Analysis and Statistics

ImageJ (1.45S, National Institutes of Health, Bethesda, USA) software was used for signal intensity measurements. For light microscopy of periodic acid Schiff’s staining, the region of interests (ROIs) including spiral ligament, spiral limbus, and stria vascularis were selected using freehand selections button. The “measure” function was used to obtain the mean gray scale value of the ROI, which was inversely correlated with the staining intensity. For confocal microscopy of immunofluorescence staining, the ROIs including stria basal cells, spiral ganglion cells, spiral ligament, and spiral limbus were extracted using photoshop CS6 (version 13.0, Adobe Systems Software Ireland Ltd, Dublin, Ireland) program and were imported into ImageJ program. The images were split into individual channel, and the green (corresponded to CD44 and TLR2) and red (corresponded to hyaluronic acid) channels were selected for further quantifications. The “Threshold” was adjusted using the “set” button in the “Image” menu, and “Limit to Threshold” option should be selected and “Direct to” should be defined to the corresponding channel in the “Analyze” menu. Then the gray scale value, which was inversely correlated with the staining intensity, was obtained using the “Measure” function in the same menu.

Statistical analyses were performed using the IBM^®^ SPSS^®^ Statistics Version 20 software package (SPSS Inc., Chicago, USA). A one-way ANOVA and Kruskal-Wallis test were used to compare ABR threshold shifts and signal intensities (grayscale) of staining for glycosaminoglycan and hyaluronic acid secretions, and TLR2 and CD44 staining between the LPN injected-ear and saline injected-ear groups in the different cochlear structures among various turns. Least significant difference (LSD) test was used as post hoc analysis. Higher numbers in the grayscale analysis correlate with lower signal intensities of the staining. *p* < 0.05 was accepted as statistically significant.

## Abbreviations

ABR: Auditory brainstem response
AgNPs: Silver nanoparticles
dH_2_O: Deionized water
GAB1: Growth factor receptor-bound protein 2-associated-binding protein 1
Gd-DOTA: Gadolinium-tetra-azacyclo-dodecane-tetra-acetic acid (DOTAREM)
Gd-MRI: Gadolinium-enhanced magnetic resonance imaging
JNK: Jun amino-terminal kinases
LPN + Gd-DOTA: Gd-DOTA-containing LPNs
LPNs: Liposome nanocarriers
ROI: Region of interests
ROS: Reactive oxygen species
TLR: Toll-like receptor
